# VisdaNet: Visual Distillation and Attention Network for Multimodal Sentiment Classification

**DOI:** 10.3390/s23020661

**Published:** 2023-01-06

**Authors:** Shangwu Hou, Gulanbaier Tuerhong, Mairidan Wushouer

**Affiliations:** Xinjiang Multilingual Information Technology Laboratory, Xinjiang Multilingual Information Technology Research Center, College of Information Science and Engineering, Xinjiang University, Urumqi 830017, China

**Keywords:** multimodal sentiment classification, knowledge distillation, visual aspect attention

## Abstract

Sentiment classification is a key task in exploring people’s opinions; improved sentiment classification can help individuals make better decisions. Social media users are increasingly using both images and text to express their opinions and share their experiences, instead of only using text in conventional social media. As a result, understanding how to fully utilize them is critical in a variety of activities, including sentiment classification. In this work, we provide a fresh multimodal sentiment classification approach: visual distillation and attention network or VisdaNet. First, this method proposes a knowledge augmentation module, which overcomes the lack of information in short text by integrating the information of image captions and short text; secondly, aimed at the information control problem in the multi-modal fusion process in the product review scene, this paper proposes a knowledge distillation based on the CLIP module to reduce the noise information of the original modalities and improve the quality of the original modal information. Finally, regarding the single-text multi-image fusion problem in the product review scene, this paper proposes visual aspect attention based on the CLIP module, which correctly models the text-image interaction relationship in special scenes and realizes feature-level fusion across modalities. The results of the experiment on the Yelp multimodal dataset reveal that our model outperforms the previous SOTA model. Furthermore, the ablation experiment results demonstrate the efficacy of various tactics in the suggested model.

## 1. Introduction

The development of information and communication technology has accelerated the integration of social media into the daily life of the public, and users have gradually transformed from traditional information consumers to information producers [1]. Content published by users on social platforms carries a large amount of personal emotion and opinion information, which is continuously spread and fermented in the virtual network, and then affects the process and development of the real world. Therefore, analyzing and identifying the latent emotional information in social data are important in the field of affective computing and natural language processing. The development of communication technology has changed the content form of traditional social data. The content published by users is no longer limited to single-text information; however, multimodal data that combine text, images, and speech encourage study into multimodal sentiment classification. This article concentrates on text and photos.

People’s decisions are heavily influenced by their sentiments. Sentiment classification seeks to automatically determine the sentiment polarity of the large number of opinions and comments appearing on the Internet, which may be binary (positive vs. negative) or ordinal along some grading scale (e.g., 1 to 5).

Different parts of a document in sentiment analysis are differentially informative, compared to neutral sentences (e.g., “I had a salad for dinner.”), emotional sentences (e.g., “Pizza is very tasty, and durian is perfect.”) may be more important. By contrast, some words (e.g., “tasty”) are more influential. These differences in information levels can be captured via attention [2], where differentiated attention is given to important content, and the corresponding sentences (or words) will be given more and higher weights.

**Figure 1:**
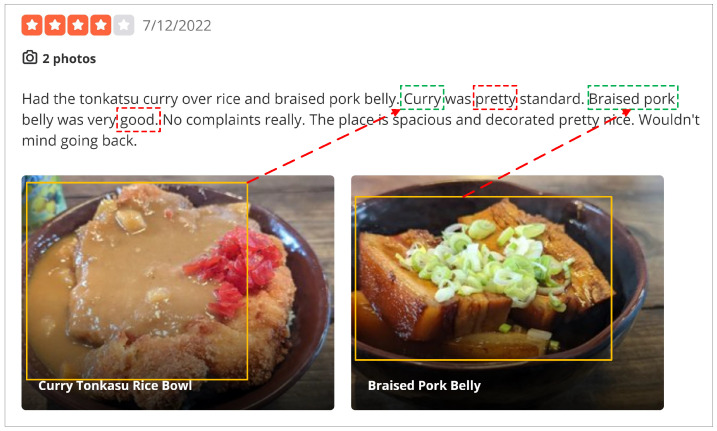
Example of text-image review. The entities in the image (yellow boxes) mark (red arrows) important entities in the text (green boxes) and direct us to focus on the adjectives that describe the entities (red boxes).

An emerging concept for sentiment classification is that in many cases images are not suitable for expressing sentiment independently of the text, but are more suitable for supporting text, highlighting salient aspects of an entity in text, and using attention to indicate the important sentences. Truong et al. [3] pointed out that visual aspect attention is more effective than visual feature or text attention. As shown in Figure 1, the “Curry” and “Braised pork” in the review text appear in the images. These entities often tend to play more important roles in judging the sentiment polarities of reviews than other words.

In the past few years, some good results have been achieved by leveraging visual modality to improve the quality of images selected by the model. For example, Zhu et al. [4] considered summary generation and image selection as training objectives, and introduced image references into the training process. This method lacks image references. To solve this problem, Zhu et al. [4] put forward a new method of ROUGE-ranking to construct pseudo-image references. Unfortunately, we find that the approach assumes that image captions have yet included condensed textual information about visual morphology, semantically matched to corresponding images. In truth, this presumption is mainly reliant on the quality of the image description rather than the content itself. However, we found that in the actual large amount of irregular data, image captions are often not as good as imagined, and sometimes even non-existent.

Based on the preceding discussion, we investigate whether it is possible to learn the relevance ranking of images from a pre-trained visual model (e.g., CLIP), so that our model can learn the relevance ranking of images in the obtained text rather than relying on the existence and quality of image captions. Fortunately, using the cosine similarity of text citations and images to represent the correlation of text-image content and utilizing this correlation to construct pseudo-image citations was discovered in the recent process of researching contrastive visual language training or CLIP (Radford et al., 2021) [5]. However, as we all know, the use of visual language models to obtain knowledge to guide multimodal sentiment analysis is still an interesting and valuable research topic.

Problem. First, traditional sentiment analysis work mostly focuses on long text content, ignoring the feature sparseness and lack of information in a short text representation, and it is difficult to learn efficient feature representation from limited words.

Second, information control problem in the multimodal fusion process. Current methods only focus on the aggregation of heterogeneous information from multiple sources, ignoring the selection and filtering of original modal long text information. In the process of multimodal sentiment expression, consistent and specific information generally exists between modalities, which requires the model to capture the complete two types of information, and filter out the noise information irrelevant to the task, so as to learn compact and efficient fusion representation.

Third, single-text multi-image fusion problem in product review scenarios. Commodity review data are usually composed of a piece of text and multiple images. These images cannot convey complete sentiment information, but can only play a role in assisting or enhancing the emotion of the text. However, the existing text-image sentiment analysis methods usually assume that text and images have the same importance, which is not in line with the actual situation of product review scenarios.

Contributions. First, this paper proposes a knowledge augmentation module, by integrating the information of image description and short text, the problem of lack of information in the short text is overcome.

Second, aimed at the problem of information control in the multimodal fusion process in the product review scene, this paper proposes knowledge distillation based on the CLIP module. This module first takes text information as the main body, uses image information to assist in locating emotion-related sentences in long texts, performs knowledge distillation on long text information, correctly models the text-image interaction relationship in special scenarios, and achieves effective cross-modality fusion while reducing the noise information of the original modal and improving the quality of the original modal information.

Third, aimed at the problem of single-text multi-image fusion in the product review scene, this paper proposes a visual aspect attention based on the CLIP module. The module first takes text information as the main body and uses image information to assist in locating emotion-related words in the text. It models the graphic interaction relationship in special scenarios and realizes the feature-level fusion of cross-modalities. Finally, in response to the above problems, we propose a visual distillation and attention network or VisdaNet for multimodal sentiment classification. The accuracy of our model on the Yelp dataset is better than the SOTA model GAFN [6].

## 2. Related Work

### 2.1. Sentiment Analysis

Traditional sentiment analysis tasks usually focus on textual content. With the development of deep learning and text classification, loading and fine-tuning the pre-trained language models have become a popular approach to obtaining the embedding representations of texts [7,8,9,10,11]. Then, attention mechanism-based models [12,13], recurrent-based neural networks [14,15], or convolution-based neural networks [16,17] could be employed to learn high-level semantic features. Kim [18] investigates top pre-trained convolutional neural network-trained word vectors for sentence-level classification problems. Lai et al. [19] firstly gives a RCNN suitable for text classification, which combines the advantages of recurrent neural networks and convolutional neural networks. Yang et al. [2] believed that different words have different importance in sentences, and different sentences also have different importance in documents. Based on this understanding, they used a hierarchical attention network on the sentiment classification task and achieved better results. Basiri et al. [20] proposed an attention-based bidirectional CNN-RNN deep model for sentiment analysis. Akhtar et al. [21] proposed a stacked ensemble approach to predict sentiment and the intensity of sentiment by combining outputs obtained from several deep learning and feature-based classical models using a multilayer perceptron network. To resolve word polysemy, Peters et al. [22] learned word embeddings by employing a learning function of a deep bidirectional language model’s internal state (biLM). Devlin et al. [23] employed a transformer [24] to obtain textual features; therefore, each word’s embedding is fused with context features. Recently, the BERT model has shown marked improvement in classification tasks. In order to solve the problem of mask corrupting input, Yang et al. [25] proposed an autoregressive pre-training method to learn bidirectional context and overcome the limitations of BERT. Valdivia et al. [26] address the ambiguity and lack of information in neutral reviews by specifying the boundary between positive and negative reviews using various weighted aggregation models to improve the model’s performance. Wang et al. [27] proposed an opinion analysis scheme with multi-level fine emotion perception with contradiction processing in order to further study the text to analyze multi-level fine emotion and different types of emotion. In [28], the authors proposed a hierarchical memory network using bidirectional gated recurrent units as utterance readers and used the fusion layer for interaction between historical utterances. Ghosal et al. [29], by using common sense knowledge, the task of discursive emotion recognition in conversation is solved. Model aspects of common sense knowledge by considering the causality of emotional identification in mental states, events, behaviors, and conversations. Li et al. [30] put forward a quick, compact, and parametric efficient square ignorance framework, as well as a bidirectional emotional recurrent unit, for sentiment analysis in the conversation.

### 2.2. Multimodal Sentiment Analysis

Multimodal sentiment analysis has gained increasing attention in recent years. text-image multimodal data are one of the most prevalent types of multimedia, particularly in product reviews. This page focuses on text and photos. Feature engineering was mostly employed in early methods of building models for text-image multimodal sentiment classification problems. Borth et al. [31], for instance, create adjective–noun pairs for image sentiment classification. The multimodal sentiment classification model that employs a neural network has been widely discussed and investigated in academic circles since the introduction of deep learning. Yu et al. [32] extracted image and text features individually and mix them for sentiment classification using pre-trained convolutional neural networks. Xu et al. [33] extracted scene and object features using two separate convolutional neural networks, then blend scene and object features with text features for multimodal sentiment classification. Xu et al. [34] updated the model again, taking into account the influence of diverse modal information, such that the various modal information can supplement each other and achieve higher performance. In [35], a multimodal hierarchical fusion model was proposed to solve the multimodal sarcasm detection task of tweets composed of text and images. Truong et al. [3] believe that many images have weak emotional expressions, and even the images themselves do not have emotions, so it is obviously unreasonable to input images as features into the classifier. Images, on the other hand, might be utilized to highlight important elements of the text. As a result, they suggested the VistaNet model, which employs the pre-trained VGG [36] network to extract picture characteristics and the attention mechanism to emphasize specific sentences in the review text, and obtains better classification results. Du et al.(2022) [6] exploited the gating mechanism to maintain relevant fine-grained visual features, which were then utilized by the cross-modal attention mechanism to emphasize the textual segment.

In recent years, deep learning has demonstrated superior performance in the field of pattern classification [37,38,39,40]. The process of human sentiment expression is a comprehensive and complementary process of various modal information. Single-modal features can only show part of the attribute information of an object. In order to describe the target object more accurately, the integration of multimodal features is an inevitable trend. Zhao et al. [37] proposed a new faster mean-shift algorithm, which solves the problem that the deployment of embedding-based algorithms is limited by the slow inference speed by introducing a new online seed optimization strategy. Yao et al. [38] proposed a simple compound figure separation (SimCFS) framework that can be efficiently deployed to new image classes without resource extensive bounding box annotation by introducing a new side loss as well as an intra-class image enhancement method. Jin et al. [39] proposed a pseudo-RGB-D face recognition framework as well as a data-driven approach for generating depth maps from 2D face images, as well as a generative adversarial network model called “D+GAN” for multi-conditional image-to-image conversion with face attributes, allowing the use of generated pseudo-depth maps instead of depth sensors. Zheng et al. [40] proposed achieving excellent image classification performance by first training a network model to extract the image representation for anomaly detection, and then retraining the network to regularize the feature boundaries based on the anomaly detection results. Wu et al. [41] suggested an end-to-end system based on edge computing to conduct image enhancement and object detection tasks in low-light environments using cloud-based enhancement and edge-based detection.

### 2.3. Knowledge Distillation

Knowledge distillation [42], which extracts knowledge from the teacher model and uses the extracted knowledge to improve the performance of the student model, usually by matching the student’s prediction with the teacher’s prediction. In practice, it is not difficult to find that most methods concentrate on acquiring knowledge from pre-trained teacher models [42,43,44,45,46], but for online distillation [47,48], we note that it is possible to train multiple models simultaneously and utilize their ensemble as a teacher. We extract knowledge from the most advanced visual language model CLIP [5] to direct our training process, which can compensate for any requirements on the presence and quality of image captions.

## 3. Visual Distillation and Attention Network

We focus on the text-image multimodal sentiment classification task. We will define the problem in this section and detail our visual distillation and attention network or VisdaNet model.

**Problem Definition.** A set of user reviews *R* are given by us. For each review r∈R, r={(txt,imgs),y}. txt represents the textual component of the review. imgs represents the visual component of the review. *y* represents the sentiment label of the review. The textual component txt is a sequence of *N* sentences $s_{n},txt=\left\{s_{n}|n\in \left[1,N\right]\right\}$, where the sentence $s_{n}$ consists of a sequence of *T* words $w_{n,t},s_{n}=\left\{w_{n,t}|t\in \left[1,T\right]\right\}$. The visual component imgs is a sequence of *H* images $i_{h}$ and image caption $c_{h}$ pairs, $imgs=\{\left(i_{h},c_{h}\right)|h\in \left[1,H\right]\}$. The text-image multimodal sentiment classification problem can also be expressed as follows: using marked labeled data *R* to train a classification function *f*, the function *f* can predict the sentiment polarity of previously unseen labeled multimodal samples containing text and images, f(txt,imgs)=y.

**Figure 2:**
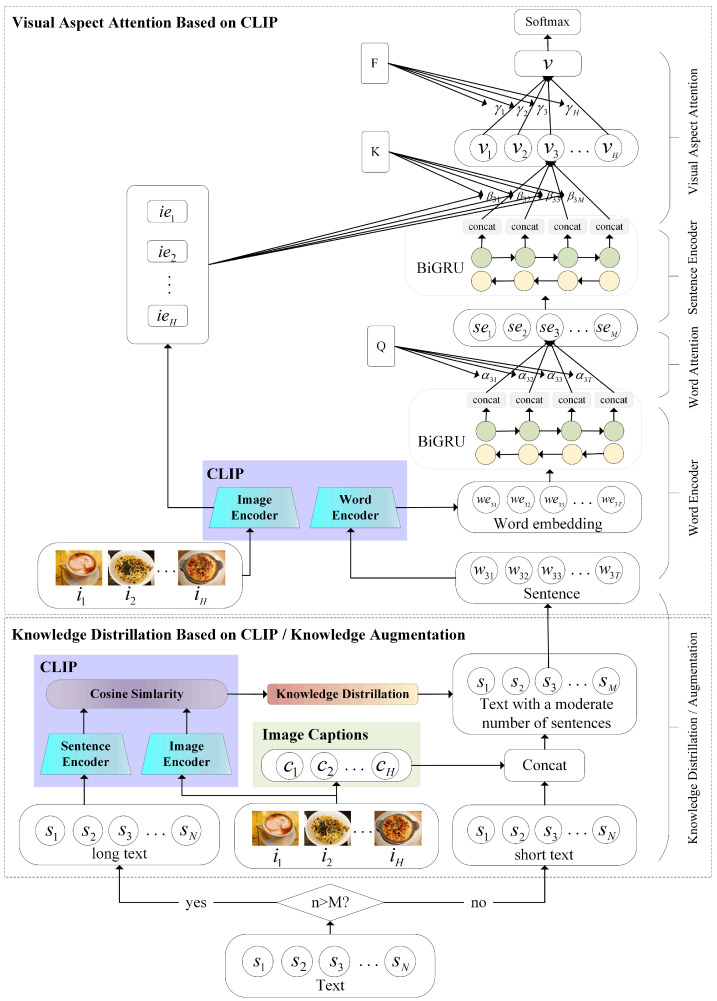
Structure of Visual Distillation and Attention Network.

VisdaNet is a hierarchical four-layer architecture as shown in Figure 2. The bottom layer is the knowledge distillation/augmentation layer, aimed at the information control problem in the multi-modal fusion process in the product review scene; long texts are shortened by the knowledge distillation module to reduce the noise information of the original modalities and improve the quality of the original modal information; short texts are augmented through the knowledge augmentation module, which overcomes the lack of information in short text by integrating the information of image captions and short text. The second layer is the word encoding layer, which converts word features into sentence features. The third layer is the sentence encoding layer; to address the single-text multi-image fusion problem in the product review scene, this layer incorporates visual aspect attention based on the CLIP module to transform sentence features into features of the entire review. The top layer is a classification layer that computes a sentiment label for user review. We will now go through each layer in greater detail.

### 3.1. Knowledge Distillation/Augmentation

Due to hardware and computing power limitations, only a limited number *M* of characters can often be processed for a single text. In the training process of a multimodal sentiment classification task, existing studies (Yang et al. [2], Truong et al. [3]) used a direct truncation strategy for text longer than *M*, while for text shorter than *M*, they used the strategy of padding the matrix with 0s. However, this strategy not only results in the loss of some sentences in long texts that are likely to be very important for sentiment polarity, but it also causes the information-poor short texts to waste the computing power of the computer.

#### 3.1.1. Knowledge Distillation

Therefore, for reviews with more than *M* sentences, we employ the knowledge distillation (KD) technique (Hinton et al. [42]) to distill text-image content correlation knowledge, which can extract knowledge from images and texts. Our proposed method aims to extract the knowledge from the teacher network CLIP (Redford et al. [5]) into the encoder of our next module, compute the text-image relevance content score, rank important sentences according to the score, and find the *M* sentences that are most relevant to emotion.

For the text component of each review, txt, we split it into sentences, and we obtain a set $s_{n}$ of length *N*.

Then, as a teaching model, we utilized CLIP (Redford et al. [5]) to compute the cosine similarity scores between sentence embeddings extracted by feeding the sentence $s_{n}$ into its text encoder T and image embeddings extracted by the feeding image collection $i_{h}$ into its image encoder I:

(1) $$S^{M}=KD\left(sim\left(T\left(s_{n}\right),I\left(i_{h}\right)\right),M\right),n\in \left[1,N\right],h\in \left[1,H\right]$$

**Figure 3:**
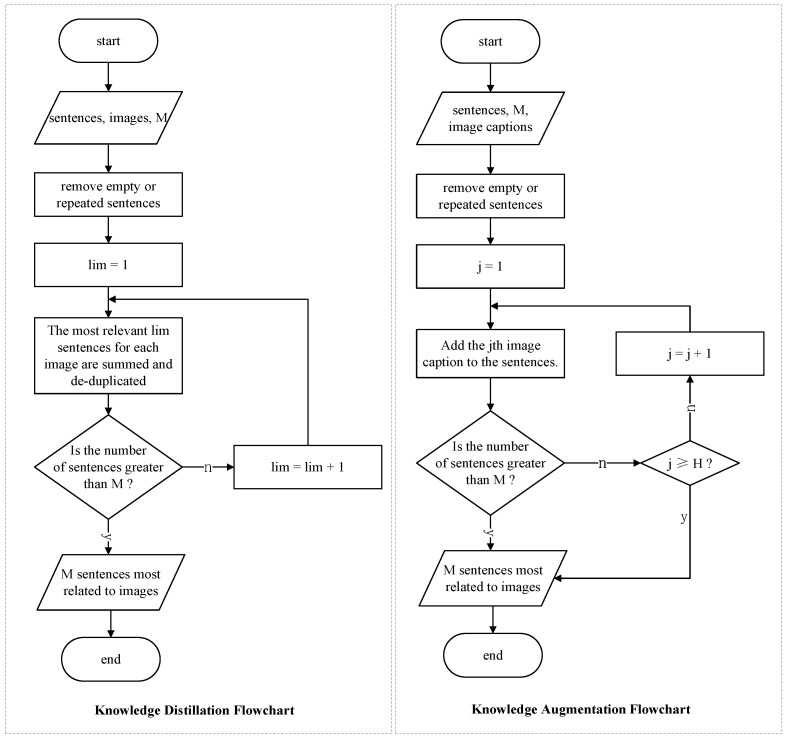
Knowledge distillation/augmentation flowchart.

The above algorithm KD description of our model is shown in Algorithm 1. The left side of Figure 3 shows the flowchart of the KD algorithm.
**Algorithm 1:** Knowledge distillation/augmentation process.
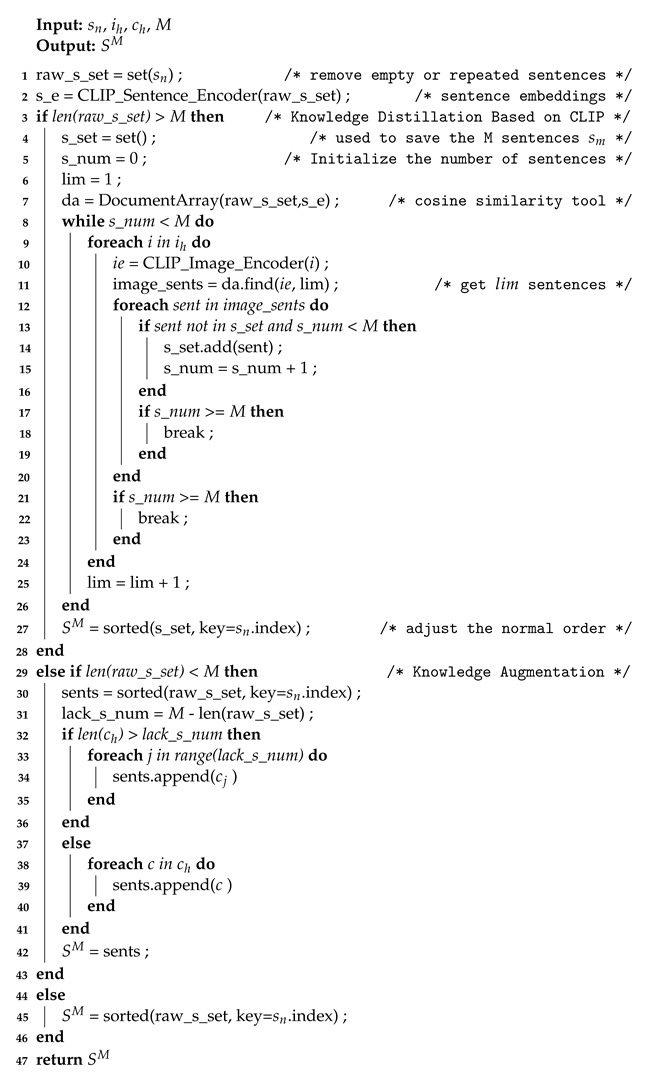


#### 3.1.2. Knowledge Augmentation

For reviews with fewer than M sentences, we perform knowledge augmentation (KA) on the text using image captions $c_{h}$:

(2) $$S^{M}=KA\left(s_{n},c_{h},M\right),n\in \left[1,N\right],h\in \left[1,H\right]$$

The above algorithm KA description of our model is shown in Algorithm 1. The core idea to perform augmentation is to achieve the effect of text expansion by contacting the native textual component (txt) with the native captions ($c_{h}$) of the images. The right side of Figure 3 shows the flowchart of the KA algorithm.

After the above Algorithm 1, we can change the sentence length of reviews from *N* to *M*, and we obtain a sentence set $S^{M},S^{M}=\left\{s_{m}|m\in \left[1,M\right]\right\}$.

### 3.2. Word Encoder with Word Attention

We split each sentence in $S^{M}$ into words $w_{m,t}$, for each word $w_{m,t}$, we use CLIP (Redford et al. [5]) to feed the word $w_{m,t}$ into its text encoder T to generate word embedding $we_{m,t}$.

(3) $$we_{m,t}=T\left(w_{m,t}\right),t\in \left[1,T\right]$$

In order to solve the problem of word order and word polysemy, the word embedding $we_{m,t}$ of the review text is then turned into a new representation $\epsilon_{m,t}$ by BiGRU [49], which is concatenated by the hidden layer vectors of the forward GRU [49] and the backward GRU [49].

(4) $${\vec{\epsilon }}_{m,t}=\vec{GRU}\left(we_{m,t}\right),t\in \left[1,T\right]$$

(5) $${\overset{\leftarrow }{\epsilon }}_{m,t}=\overset{\leftarrow }{GRU}\left(we_{m,t}\right),t\in \left[T,1\right]$$

(6) $$\epsilon_{m,t}=\left[{\vec{\epsilon }}_{m,t};{\overset{\leftarrow }{\epsilon }}_{m,t}\right]$$

In a sentence, not all words have equal contribution to the expression of the sentence sentiment, some words have stronger sentiment information, and these words are more important for sentiment detection. Therefore, when the word representations of a sentence are aggregated into sentence representations, we use the word attention mechanism to learn and assign a weight *Q* among words corresponding to their “importance” in the sentence representation.

(7) $$q_{m,t}=tanh\left(W_{q}\epsilon_{m,t}+b_{q}\right)$$

(8) $$\delta_{m,t}=Q^{T}q_{m,t}$$

(9) $$\alpha_{m,t}=\frac{exp(\delta_{m,t})}{\sum_{t}^{}exp\left(\delta_{m,t}\right)}$$

(10) $$se_{m}=\sum_{t}^{}\alpha_{m,t}\epsilon_{m,t}$$

In more detail, we first input the word representation $\epsilon_{m,t}$ through a single-layer MLP with a nonlinear activation function tanh, to obtain $q_{m,t}$ as the hidden representation of $\epsilon_{m,t}$, and then multiply the hidden representation $q_{m,t}$ with a context vector $Q^{T}$ (initialized at random and learned during training) to obtain the similarity $\delta_{m,t}$ of $q_{m,t}$, and $Q^{T}$, which is a scalar. We use this scalar $\delta_{m,t}$ to represent the relative importance of words in a sentence, and normalize it by a softmax function to produce a normalized attention weight $\alpha_{m,t}$. Finally, we obtain the sentence embedding $se_{m}$ by the weighted summation of all the word representations $\epsilon_{m,t}$ and attention weights $\alpha_{m,t}$.

### 3.3. Sentence Encoder with Visual Aspect Attention Based on CLIP

In order to solve the problem of sentence order, given the sentence embedding $se_{m}$, we can obtain a new review representation $\epsilon_{m}$ in a similar way. The sentences are encoded using a bidirectional GRU:

(11) $${\vec{\epsilon }}_{m}=\vec{GRU}\left(se_{m}\right),m\in \left[1,M\right]$$

(12) $${\overset{\leftarrow }{\epsilon }}_{m}=\overset{\leftarrow }{GRU}\left(se_{m}\right),m\in \left[M,1\right]$$

(13) $$\epsilon_{m}=\left[{\vec{\epsilon }}_{m};{\overset{\leftarrow }{\epsilon }}_{m}\right]$$

The use of a text-based soft attention pooling approach (Yang et al. [2]) is one option for obtaining the final representation *v* of the review *r*. Truong et al. [3], on the other hand, support the use of visual information to improve attentional mechanisms. Multiple photos that correspond to various “aspects” may each have an associated review. Sentences can contain varying quantities of information given an image. In other words, the visuals will draw attention to various yet significant sections of the review. In order to enhance the quality of the learned review representation *v*, we want to use this soft attention technique with visual information. We follow Truong et al. [3] and call this the visual aspect of attention.

The input photos need to be encoded first. For various image-related tasks, CLIP (Radford et al. [3]) is an effective and scalable method for learning SOTA picture representations from scratch. By feeding the picture $i_{h}$ through the model and then obtaining the output after CLIP, we may utilize CLIP to obtain a representation of the image $ie_{h}$. By encoding the image $i_{h}$, a 512-dimensional vector known as the image representation $ie_{h}$ is created.

(14) $$ie_{h}=CLIP\left(i_{h}\right),h\in \left[1,H\right]$$

For each image representation $ie_{h}$, we learn the attention weights $\beta_{h,m}$ of the sentence representation $\epsilon_{m}$.

(15) $$d_{h}=tanh\left(W_{d}ie_{h}+b_{d}\right)$$

(16) $$g_{m}=tanh\left(W_{g}\epsilon_{m}+b_{g}\right)$$

(17) $$k_{h,m}=d_{h}⊙g_{m}+g_{m}$$

(18) $$\lambda_{h,m}=K^{T}k_{h,m}$$

(19) $$\beta_{h,m}=\frac{exp(\lambda_{h,m})}{\sum_{m}^{}exp\left(\lambda_{h,m}\right)}$$

We first project the sentence representation $\epsilon_{m}$ and the image representation $ie_{h}$ onto an attention space, and then we use a non-linear activation function to determine the outputs, which are $d_{h}$ and $g_{m}$, respectively, to learn these attention weights. To ensure that neither component of the activation function dominates the other, we use tanh to scale $ie_{h}$ and $\epsilon_{m}$ into the same range of values. For a reason that will be explored in more detail later, we allow the image projection $d_{h}$ to interact with the sentence projection $g_{m}$ in two different ways: element-wise multiplication and summing. This allows us to determine the picture-specific attention weight of a phrase. Similar to how *Q* functions at the word level, the learned vector $K^{T}$ serves as the context for global attention. This generates an attention value $\lambda_{h,m}$ that is then normalized $\beta_{h,m}$ using softmax.

$k_{h,m}$ must be calculated using both element-wise multiplication and summing to ensure that the image and sentence interact meaningfully. When computing attention weight $\beta_{h,m}$ without element-wise multiplication and just summing, the softmax function would have eliminated the influence of the visual component. Due to the absence of visual material, the impact of the text segment would have been substantially lessened without the summing and with only element-wise multiplication. As a result, both are required for effective visual aspect attention. If we remove $g_{m}$ from Equation (17), our proposed mechanism may be shown to step across “bilinear” attention (Kim et al. [50]), which has greater interactions than “concat-product” attention (Bahdanau et al. [51]).

The sentence representations $\epsilon_{m}$ are aggregated into an image-specific review representation $v_{h}$ using the image-specific attention weights $\beta_{h,m}$.

(20) $$v_{h}=\sum_{m}^{}\beta_{h,m}\epsilon_{m}$$

We use this visual aspect attention technique for a review and apply it to each of the images, producing a collection of aspect-specific review representations $v_{h},hin\left[1,H\right]$. Before categorization, all of the $v_{h}$s must be combined into the final review representation *v*. Images are differently instructive when given an evaluation. In order to determine how each image-specific review representation $v_{h}$ will affect the ultimate review representation *v*, we want to determine the important weight $\gamma_{h}$.

(21) $$f_{h}=tanh\left(W_{f}v_{h}+b_{f}\right)$$

(22) $$\theta_{h}=F^{T}f_{h}$$

(23) $$\gamma_{h}=\frac{exp(\theta_{h})}{\sum_{h}^{}exp\left(\theta_{h}\right)}$$

Through a layer of neurons with nonlinear activation function tanh, aspect-specific review representation $v_{h}$ is projected into attention space. The global attention context vector $F^{T}$ (initialized at random and learned during training) is multiplied by the scalar $f_{h}$ to determine the relevance of $v_{h}$. According to Figure 2, utilizing soft attention pooling and review-to-image attention weights ($\gamma_{h}$), the review representations $v_{h}$ owing to the various pictures are combined into the final review representation *v*.

(24) $$v=\sum_{h}^{}\gamma_{h}v_{h}$$

### 3.4. Sentiment Classification

In the top layer, we then use the high-level representation of the review *v* to produce the probability distribution over classes η by treating it as features for a softmax-based sentiment classifier.

(25) $$\eta =softmax(W_{r}v+b_{r})$$

By reducing the sentiment classification cross entropy error, the model is trained under supervision.

(26) $$loss=-\sum_{l=1}^{L}ylog\eta \left(l\right)$$

where *y* denotes the ground truth label, *L* denotes the number of sentiment polarity categories, and η(l) is the likelihood that the sentiment polarity will be forecasted as the *l*th category.

## 4. Experiment and Analysis

This section conducts comprehensive experiments to evaluate the effectiveness of knowledge augmentation, knowledge distillation based on CLIP, and visual aspect attention based on CLIP. All code is written in Python 3.6 on Ubuntu 18.04.9, and the framework for deep learning is TensorFlow 1.14.0. The training process was accelerated using an Intel Core i9-9900K CPU @ 3.6 GHz × 16 and a GeForce RTX 3090 GPU.

### 4.1. Dataset and Experimental Settings

The Yelp restaurant review dataset [3], which was acquired from the Yelp restaurant review website and contains text-image pairings of restaurant reviews from five American cities, was used for the experiments. The review text was lengthy and contained numerous sentences in it. In almost all reviews, there were three or more images. The sentiment polarity label was determined by the reviews’ ratings in the dataset. The user was more satisfied with a higher rating. There were exactly the same amounts of samples in each of the five categories. Table 1 displays the dataset’s statistical information. A total of 44,305 samples were used, and they were split into training, validation, and test sets at the ratios of 8:0.5:1.5. A test set was split into five subsets based on the locations of the restaurants: Boston (BO), Chicago (CH), Los Angeles (LA), New York (NY), and San Francisco (SF).

We utilized NLTK (Loperand et al. [52]) for preprocessing to tokenize sentences and words. We create vocabularies out of terms that appear more than three times in both the training and validation sets, and we switch out other words that are only sometimes used with unique UNK tokens. A pre-trained CLIP [5] vector with 512 dimensions is used to initialize the word embeddings. Throughout the training, the word embeddings are adjusted. Unknown words have uniform distributions U[−0.01, 0.01] that are initialized at random. *M* (the number of sentences in each review) is a key hyperparameter. We detail the basis for setting the model hyperparameters in the following.We take the value of *M* as 30.

Hyperparameters are used to fine-tune each model to achieve the best results on the validation set. The GRU cells have 50 dimensions for encoding words and sentences (due to the bidirectional GRU 100-dimensional). The attention spaces of the word, sentence, and review have 100-dimensional context vectors Q, K, and F as well. For feature extraction from images, we employ CLIP [5]. With a minimum batch size of 32, we employ RMSprop (Tieleman et al. [53]) for gradient-based optimization during training. To avoid over-fitting, the dropout rate is set to 0.5. Table 2 lists the other experiment‘s hyperparameters. In Table 3, we present the influence of the value of the hyperparameter *M* (number of sentences in each review) on the model classification performance. As we can see, the model prediction accuracy is highest at M=35, but in the comparison experiments in this paper, we set *M* to 30 to ensure fairness in the experimental comparison with the SOTA model GAFN [6] and the model VistaNet [3]. Similarly, we set *T* (number of words in each sentence) to 30 to ensure fairness for experimental comparisons with the SOTA model GAFN [6] and the model VistaNet [3]. In Table 4, we present the influence of the value of the hyperparameter *H* (number of images per review) on the model classification performance. We discover a common tendency in which classification accuracy increases as the number of images increases. Since each review in the dataset has at least 3 images, we set the value of *H* to 3 in order to make full use of each review and maximize the use of image information. After several iterations of each comparison approach, we fine-tune the model hyperparameters on the validation set and present the average findings using statistical testing on the test set. TensorFlow is employed to implement VisdaNet.

### 4.2. Comparison Experiment

To compare the effectiveness of the model suggested in this research, we chose the following traditional approaches to the task of sentiment classification.

- TextCNN [18]: Convolutional neural networks are used by Kim et al. [18] to extract text features, which can help predict sentiment polarity by capturing key information from the text. Additionally, TextCNN_CLIP concatenates the text representation with the image feature representation for classification by using the CLIP [5] model to extract the image feature representation.
- FastText [54]: It was suggested by Bojanowski et al. [54] to add sub-word information to word representations. Its network architecture is straightforward, but it performs well when it comes to text classification. In order to compare it to BERT [23], it is used to create word embedding representations.
- BiGRU [55]: Tang et al. [55], the employment of gating mechanisms to address the sequence modeling issue of long-distance dependence, which results in improved quality text representation. In order to extract the features of the images and combine them with the text representation for classification, BiGRU_CLIP also employs the CLIP [5] model.
- HAN [2]: Yang et al. [2] suggested a hierarchical attention network. Before producing a representation of the text at the document level, it takes into account the significance of various words in sentences as well as the significance of various sentences within the document. In order to combine the text representation with the picture features representation for classification, HAN_CLIP additionally employs the CLIP [5] model.
- BERT [23]: A extremely long-term dependence based on multi-head attention can be captured by the pre-trained language model put forth by Devlin et al. [23]. A train set’s textual contents are used to fine-tune BERT for sequential classification tasks.
- VistaNet [3]: A multimodal sentiment classification network based on HAN [2] is proposed by Truong et al. [3] and employs visual features to weight sentence representation.
- GAFN [6]: Du et al. [6] adopt a gated attention method to integrate visual and text information, allowing them to not only fully utilize multimodal information but also mitigate the influence of noise image.
- VisdaNet(Ours): The model proposed in this paper makes full use of multimodal information for knowledge supplementation of short texts as well as knowledge distillation of long texts, which can, at the same time, solve the problem of feature sparsity and information scarcity in short text representation and filter the task-irrelevant noise information in long texts.

On the dataset, the performance of our proposed model and the aforementioned baseline methods for sentiment classification is compared. Accuracy values are used to evaluate it. The essential characteristics of each method are listed in Table 5. The results of the experiment are displayed in Table 6.

Interestingly, the TextCNN model works well in the field of text sentiment classification, come out to perform the worst among compared approaches, with accuracy values for TextCNN and TextCNN-CLIP of 53.88% and 54.34%, respectively.

The accuracy of BiGRU is 56.52%. BiGRU-CLIP increases accuracy by combining image features, but only by 0.4% compared to BiGRU and by 4.4% compared to TextCNN-CLIP. These models use concatenation to combine elements from the review text and the images.

In comparison to BiGRU and BiGRU-CLIP, hierarchical HAN and HAN-CLIP perform better. When combining image features, HAN-CLIP performs somewhat better than HAN 57.33%, coming in at 59.19%. When contrasted to BiGRU, the text module’s hierarchical modeling is what makes these advances.

**Figure 4:**
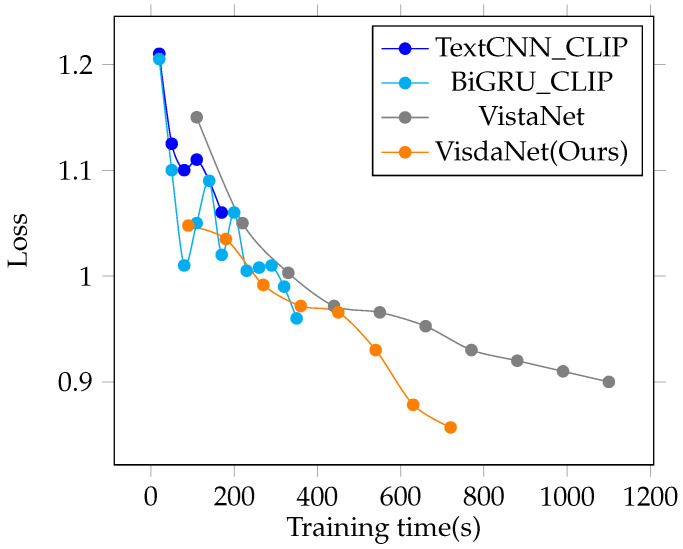
Comparison of the model loss value and training time change.

Figure 4 displays the loss value as the train set’s training time changes. Figure 4 and Table 3 can reflect the complexity and processing speed of the proposed model. In terms of the single-epoch training time, the number of convergence epochs, and loss value, the suggested VisdaNet model surpassed VistaNet.

VisdaNet, the model suggested in this paper, clearly performs best in terms of average accuracy. It outperforms the previous SOTA model GAFN by about 4%. Moreover, our model has the best accuracy performance on the largest scale Los Angeles data, outperforming the previous SOTA model GAFN by approximately 6%.

It can be seen that VisdaNet, the model proposed in this paper, has the best average accuracy performance on the Yelp dataset, improving the results by about 4% over the previous model VistaNet. In contrast to the VistaNet model, our model uses not only a hierarchical attention structure but also a knowledge distillation module and an augmentation module. The results support our hypothesis that image information can effectively solve the information scarcity problem for short texts, as well as the information control problem for long texts. By simply truncating the long text directly and ignoring the short text, it makes it difficult for the model to find useful text–visual alignments for sentiment because the short text is information-poor, the discarded parts of the long text have a lot of important information, and the parts left behind contain some useless information that should be discarded more.

### 4.3. Ablation Experiment

We conducted ablation experiments, which started with the entire architecture and gradually removed components to arrive at the simplest configuration (to investigate the relative contributions of the different VisdaNet architectural components). The results are displayed in Table 7.

- VisdaNet(Full Model): The complete visual distillation and attention mechanism model proposed in this paper.
-KnDist: The model removes knowledge distillation based on the CLIP module.
-KnAug: The model removes the knowledge augmentation module.
-ViAspAttn: The model removes the visual aspect attention based on CLIP.
-WordAttn: The model removes the word attention layer. It is a hierarchical structure of reviews.
-HiStruct: The model removes the hierarchical structure of the reviews. It is a base model (BiGRU) relying only on text.
-BiGRU+Es: Replace the VisdaNet’s text feature extraction module BiGRU with ELECTRA-small [56].

In the ablation experiments, the entire model produced the best results. The performance was statistically significant on the mean and throughout the five cities when compared to the benchmark model. In comparison to the second-best model, the results remain considerable in the means across four cities. These findings corroborate the idea that each component of the VisdaNet design contributes to the overall performance of the model. The information scarcity problem of short text was overcome by merging the information of image description with a short text. We also use image information to help locate emotion-related sentences in long texts, distill the knowledge of long text information, correctly model graphical–textual interactions in special scenarios, and achieve effective cross-modal fusion while reducing noise information and improving the quality of the original modality information.

### 4.4. Knowledge Distillation Based on CLIP Visualization

**Figure 5:**
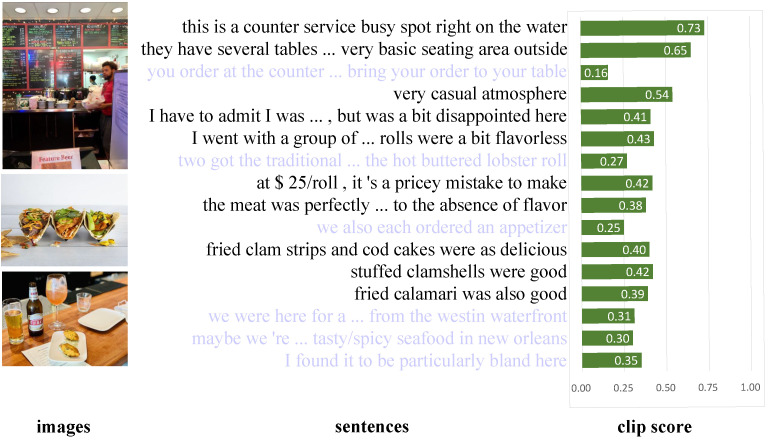
A sample of knowledge distillation based on the CLIP visualization. For the sake of simplicity, here we take an example of M=10.

Figure 5 shows an example of knowledge distillation based on CLIP visualization. The CLIP score is the cosine similarity between the sentence embedding and the image embedding. It can be seen that the emotional importance of the sentence is highly correlated with the text-image correlation.

### 4.5. Illustrative Examples

Here are a number of exemplary instances to provide some intuitive awareness for how the visual aspect attention based on CLIP may operate to improve the effectiveness of VisdaNet.

**Figure 6:**
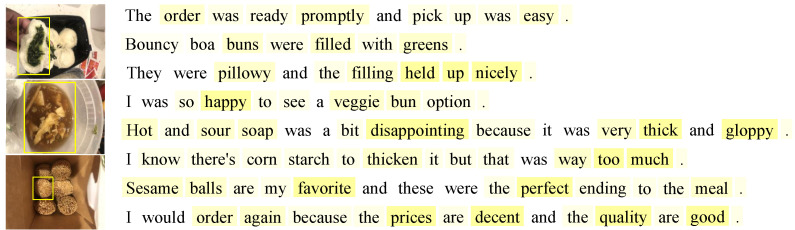
A review from a Chinese restaurant in San Francisco from Yelp.com with a rating of 4. The color depth represents the weight proportional to the importance of the sentiment.

Figure 6 depicts an example of a four-star rating. Its three images are shown on the left. The review sentences obtained after the knowledge distillation module is shown on the right in the same sequence as the original one. VisdaNet’s word attention mechanism highlights specific words in each sentence, with deeper colors representing higher attention weights. Moreover, each image emphasizes some crucial aspects to aid in word-level and sentence-level attention weighting.

The bun is depicted visually in the first image. The image emphasizes the second line “filled with greens” the third sentence “the filling held up nicely” and the fourth sentence “so happy”, which displays pleasant thoughts about the dish. The second image portrays a soup and emphasizes the fifth sentence “disappointing” and the sixth sentence “but … way too much” which indicates a strong negative sentiment about food. The third image shows some sesame balls, and the image focuses on the seventh sentence, “my favorite … perfect ending” which indicates a strong favorable sentiment about food. The self-learning derived weighted marker “promptly … easy” in the first sentence and “order again … prices are decent … quality are good” in the eighth sentence improved the likelihood that the review sentiment was positive. We can see from the highlighted words that “nicely”, “happy”, “favorite”, “perfect” and “disappointing” are more highly regarded than other terms in the review. This is another indication of the restaurant’s positive sentiment, despite its small shortcomings.

## 5. Conclusions

We propose a novel text-image multimodal sentiment classification approach based on visual distillation and attention mechanism. The model has a four-layer architecture; extra long texts are shortened and short texts are augmented. Word features are converted into sentence features, sentence features are transformed into features of the entire review, and finally, a sentiment label is computed for user review. Based on the problem of missing information in short texts and the existence of noisy information in long texts, we designed a knowledge augmentation module and CLIP-based knowledge distillation module to improve the quality of the original modal information. Finally, based on the natural alignment performance of text features and image features obtained by CLIP, we propose a CLIP-based visual aspect attention module for the single-text multi-image fusion problem in product review scenarios to correctly model the text-image interaction relationship in special scenarios and achieve cross-modal feature-level fusion. The comparative trials show that our model outperforms other relevant models on the Yelp multimodal datasets. The ablation trials confirm the usefulness of the model’s various tactics.

## Figures and Tables

**Table 1:** Statistics of the Yelp dataset.

Datasets	City	#Reviews	Avg. #s	Max. #s	Avg. #w	Min. #w	Max. #w	#Images	Min. #Images
Train	-	35,435	14.8	104	225	10	1134	196,280	3
Valid	-	2215	14.8	104	226	12	1145	11,851	3
	BO	315	13.4	85	211	14	1099	1654	3
	CH	325	13.5	96	208	15	1095	1820	3
Test	LA	3730	14.4	104	223	12	1103	20,254	3
	NY	1715	13.4	95	219	14	1080	9467	3
	SF	570	14.8	98	244	10	1116	3243	3
Total	-	44,305	14.8	104	237.3	10	1145	244,569	3

**Table 2:** Settings of the hyperparameters.

Hyperparameters	Settings
optimizer	RMSprop
learning rate	0.001
batch size	32
dropout rate	0.5
image representation dimension	512
sentence representation dimension	512
word representation dimension	512
GRU representation dimension	50
bidirectional GRU representation dimension	100
attention dimensions	100
*M* (number of sentences in each review)	30
*T* (number of words in each sentence)	30
*H* (number of images per review)	3
number of classes of prediction	5

**Table 3:** The influence of hyperparameter *M* (number of sentences in each review) on the model classification performance (Accuracy).

*M*	Boston	Chicago	Los Angeles	New York	San Francisco	Mean	Time Cost (s) *
20	64.13	66.15	60.80	61.81	58.77	61.31	519
25	64.44	65.23	61.26	60.41	60.88	61.35	627
30	62.86	62.77	**62.57**	**62.10**	60.70	62.32	720
35	**65.48**	**67.38**	62.25	61.92	**60.88**	**62.45**	1016
40	58.10	63.88	60.64	60.23	58.87	60.32	1214

* Time cost indicates the time required to train the optimal model on the training and validation sets. The unit is seconds (s).

**Table 4:** The influence of hyperparameter *H* (number of images per review) on the model classification performance (Accuracy).

*H*	Mean Accuracy
1	61.57
2	62.04
3	62.32

**Table 5:** Structure comparison to multimodal baselines.

Model	Textual Features	Visual Features	Hierarchical Structure	Visual Aspect Attention	Knowledge Distillation/Augmentation
TextCNN	✓	-	-	-	-
TextCNN_CLIP	✓	✓	-	-	-
FastText	✓	-	-	-	-
BiGRU	✓	-	-	-	-
BiGRU_CLIP	✓	✓	-	-	-
HAN	✓	-	✓	-	-
HAN_CLIP	✓	✓	✓	-	-
BERT	✓	-	-	-	-
VistaNet	✓	✓	✓	✓	-
GAFN	✓	✓	-	✓	-
VisdaNet(Ours)	✓	✓	✓	✓	✓

**Table 6:** Performance comparison to baselines on the Yelp restaurant review dataset (Accuracy).

Model	Boston	Chicago	Los Angeles	New York	San Francisco	Mean
TextCNN	54.32	54.80	54.03	53.58	53.04	53.88
TextCNN_CLIP	55.61	55.45	54.36	54.16	53.47	54.34
FastText	61.27	59.38	55.49	56.15	55.44	56.12
BiGRU	54.94	56.02	56.45	58.27	52.80	56.52
BiGRU_CLIP	58.69	57.24	56.60	57.02	55.48	56.74
HAN	61.60	58.53	57.61	57.14	53.02	57.33
HAN_CLIP	62.22	62.15	58.45	59.77	58.95	59.19
BERT	60.13	60.71	59.17	58.89	60.24	59.31
VistaNet	**63.17**	63.08	59.95	58.72	59.65	59.91
GAFN	61.60 *	**66.20** *	59.00 *	61.00 *	60.70 *	60.10 *
VisdaNet (Ours)	62.86	62.77	**62.57**	**62.10**	**60.70**	**62.32**

* Represents data we obtained from the original paper as published by the original authors.

**Table 7:** Comparison results in architecture ablation analysis experiments (Accuracy).

Model	Boston	Chicago	Los Angeles	New York	San Francisco	Mean
VisdaNet (Full Model)	**62.86**	62.77	**62.57**	**62.10**	**60.70**	**62.32**
-KnDist	62.54	**64.62**	61.96	61.92	60.53	61.98
-KnAug	61.90	61.54	60.99	59.53	59.30	60.54
-ViAspAttn	62.38	63.47	59.65	58.85	57.34	59.56
-WordAttn	59.39	63.39	58.08	58.58	58.18	58.54
-HiStruct	56.70	59.01	55.74	55.59	54.84	55.83
-BiGRU+Es	53.41	55.70	54.89	55.78	54.13	55.02

## Data Availability

The datasets involved in this study are available in publicly accessible repositories. The Yelp dataset can be found at https://github.com/PreferredAI/vista-net, accessed on 24 November 2021.
